# When Doctors Differ Where Is Safety?

**Published:** 1871-05

**Authors:** 


### Correspondence.

### WHEN DOCTORS DIFFER WHERE IS SAFETY?

In the columns of THE EXAMINER for January, March, and April, the subject of "Morphia as a Parturient" has been treated by different members of the profession, and results of experience and observation differing so widely and so antagonistically with each other, have been expressed by men of acknowledged ability and success as practitioners, that the question has become one of embarrassment, especially to our young men just entering upon the arduous duties of practice.

In my early professional training, I was taught to receive the doctrine of *specific remedial agency* with much caution and many grains of allowance, and in the course of a somewhat protracted experience, I have proven, time after time, that those teachings were wise and judicious.

The nearest approach to *specific agency*, perhaps, is that of sulphate quinine in intermittents, and sulphur in scabies, both of which do occasionally fail, and the physician prescribing is doomed to disappointment. An occasional failure, however, to meet the demand and promptly and at once fulfil the indications, is scarcely, I think, convincing evidence that the remedy is at fault, or possesses properties, other than those under the light of science and experience ascribed to it, but rather that some pathological or other condition of the case, not apparent at the time, was the preventing cause, so that, in that particular case, the usual effects of the remedy were not realized.

With regard to the subject before us, we have two medical agents or articles of medicine, differing widely in both their history and properties, and so far as experience in their uses go, produce opposite effects when administered to the parturient female. At least, in the light of passed experience, such has been the generally received opinion of the profession.

The sulphate morphia, while not regarded as an anæsthetic, is, by the profession generally, considered as justly entitled to head the list of anodynes, and as such, has been, and still is, one of our most valuable agents. Testimony in its favor, so voluminous and convincing, scarcely admits of a doubt, especially in that peculiar condition of the *encient* female, upon which abortion is so often made to depend.

But even under these circumstances, it not unfrequently happens, that under the most judicious use of the agent, the abnormal process continues, and abortion is the result, not, however, as the effect of the remedy, but in spite of its use; depending, in some cases, upon causes known and well understood, and, in other cases, the cause is so couched in obscurity, that the fact must be accepted, though hard to be explained.

I have not, at any time, because of a failure to realize the soothing and anodyne effects common to its use under these circumstances, felt authorized to attribute new and opposite results to sulphate morphia; and, although the process of abortion or labor at term may go forward to completion, even with increasing energy, while morphia is being given, it only proves to my mind, that nature, in the use of her own means, is disposed to accomplish her own work in her own good time and way.

